# Exploring the influence of personal factors on physiological responses to mental imagery in sport

**DOI:** 10.1038/s41598-023-29811-6

**Published:** 2023-02-14

**Authors:** Dagmara Budnik-Przybylska, Paweł Syty, Maria Kaźmierczak, Marta Łabuda, Łukasz Doliński, Adrian Kastrau, Patryk Jasik, Jacek Przybylski, Selenia di Fronso, Maurizio Bertollo

**Affiliations:** 1 Division of Sport Psychology, Institute of Psychology, Faculty of Social Science, University of Gdańsk, Gdańsk, Poland; 2 Institute of Physics and Applied Computer Science, Faculty of Applied Physics and Mathematics, Gdańsk University of Technology, Gdańsk, Poland; 3 BioTechMed Center, Gdańsk University of Technology, Gdańsk, Poland; 4 Division of Family Studies and Quality of Life, Institute of Psychology, Faculty of Social Sciences, University of Gdańsk, Gdańsk, Poland; 5 Department of Biomechatronics, Faculty of Electrical and Control Engineering, Gdańsk University of Technology, Gdańsk, Poland; 6 Department of Medicine and Aging Sciences, Behavioral Imaging and Neural Dynamics Center, University “G. d’Annunzio” of Chieti-Pescara, Chieti, Italy

**Keywords:** Biophysics, Physiology, Psychology

## Abstract

Imagery is a well-known technique in mental training which improves performance efficiency and influences physiological arousal. One of the biomarkers indicating the amount of physiological arousal is skin conductance level (SCL). The aim of our study is to understand how individual differences in personality (e.g. neuroticism), general imagery and situational sport anxiety are linked to arousal measuring with SCL in situational imagery. Thirty participants aged between 14 and 42 years (*M* = 22.93; *SD* = 5.24), with sport experience ranging between 2 and 20 years (*M* = 10.15; *SD* = 4.75), took part in our study. Participants listened to each previously recorded script and then were asked to imagine the scene for a minute. During the task SCL was monitored using the Biofeedback Expert 2000. Machine learning predictive models based on artificial neural networks have been trained for prediction of physiological response, as a function of selected psychological tests. We found an association among neuroticism, prestart anxiety, and general tendency to use imagery with SCL. From a practical point of view our results may help athletes, coaches, and psychologists to be more aware of the role of individual differences in sport.

## Introduction

Imagery is a multimodal cognitive simulation process that enables us to represent perceptual information in our minds in the absence of actual sensory input. It is also a well-known technique in mental training that, aside from its behavioral effect on performance, can also influence physiological arousal^1–3^. In his bioinformational theory, Lang^4–6^ emphasized the role of stimuli, that is, characteristics of the imagined situation (e.g., starting in a competition), in eliciting certain physiological responses such as increased heart rate or skin conductance level (SCL) in individuals. This autonomic response^2^ depends on the activation of coded information stored in long-term memory, where abstract units of information organized into three categories of information are stored (i.e., stimulus, response and meaning propositions). Among the possible predictors of the level of psychophysiological changes during imagery^7^ we can list factors like individual higher levels of imagery ability or the extent to which scenes are personally relevant and known to the subject, as well as an image script of the situation and direct instructions to experience the image physiologically.

A reliable biomarker of psychophysiological activation is electrodermal activity (EDA), both for actual and imagined situations. The term EDA is used to describe the whole range of phenomena closely related to autonomic emotional and cognitive processing, which are manifested by changes in the electrical properties of the skin. The independence of EDA from parasympathetic activity makes it widely used, as an indicator of emotional processing and sympathetic activity^8^. A general measure of EDA is skin conductance (SC), which shows fluctuations due to the appearance of sweat on the skin, secreted in response to activation of the autonomic nervous system (ANS). Two main components of SC can be distinguished, which differ in their dynamics of fluctuation. The first component is the skin conductance level (SCL), presented as the average of all SC measurement data recorded over a given signal time window. Changes in SCL can become apparent within several dozen seconds of the measurement duration and are thought to reflect the overall changes in autonomic arousal. SCL is usually recommended when the monitoring lasts longer. The second component is the phase component, the Skin Conductance Response (SCR), and refers to the faster changing components of the signal, which is arousal from a given stimulus. The change in SCR is recorded each time the participant experiences an emotional change^9^.

The question arises whether personal or contextual factors can explain the link between imagery and physiological responses. Bioinformational theory^4–6^ could be a cognitive explanation for the psychophysiological mechanisms of mental imagery, but without a direct reference to individual differences. Moving forward, in their revised applied model of deliberate imagery use (RAMDIU)^10^ Cumming and Williams included the component ‘who (individual)’, which embraced individual characteristics like gender, competitive level, age, experience, personality and temperament. This model incorporated characteristics of the individual (“who”), and distinguishes between “what” is imaged (i.e. the content) and “why” (i.e. the function) it is imaged by recognizing the importance of personal meaning. RAMDIU also expands how imagery ability is likely to influence the relationship between imagery use and outcomes.

Previous research showed that SCL increased during positive and negative imagery^11^ or illness, with fearful and joyful imagery compared to neutral imagery^12^, suggesting high levels of arousal as a manifestation of mental and behavioral reactions to stimuli. Neutral imagery tended to reduce arousal below the original baseline levels^11^. However, mental imagery might be viewed as the emotional amplifier of the arousal response^13^. The inability to visualize imagery scenarios, such as in people without visual imagery (aphantasia), was also linked to a lower SCL than in the control group while imaging frightening stimuli^14,15^. A study by Hägni et al.^16^ confirmed that audio-visual stimulation combined with mental imagery induced higher skin conductance after a sudden threat to a virtual limb, compared to audio-visual stimulation alone.

Since the aforementioned ‘who’ components also referred to individual differences in temperament, the concept of general arousal to stimuli or trait arousability^17,18^ should be explained. In referring to temperamental traits, Eysenck^19,20^ compared extraverts and emotionally stable individuals with introverted and neurotic individuals, and concluded that the latter showed increased cortical and autonomic arousal to stimuli^21^. More precisely neurotic individuals generally showed increased reactivity of the limbic system and strong but also long-lasting reactions to emotional experiences, which were reflected in physiological responses like heart rate or electrodermal activity. The biological theory of personality also evidenced that people are characterized by high and low arousability, which is treated as a trait. More arousable individuals might thus be more reactive to external stimuli and invest more imagery and emotions in the internal processing of their own experiences^22^.

Eysenck claimed that high neuroticism was linked to increased reactivity of the limbic system and a strong but also long-lasting reaction to emotional experiences which can also be observed in physiological responses like heart rate or electrodermal activity. Moreover, in Costa and McCrae’s personality theory, neuroticism is associated with negative emotionality and a higher predisposition to anxiety^23^.

What is highly evidenced is the relationship between emotions and personality, with a specific focus mainly on extraversion and neuroticism^23^. Individuals with higher levels of neuroticism exhibit both a greater skin conductance reactivity to emotional (and particularly aversive) pictures and more extended reactivity than emotionally stable individuals^24^. A study by Cruz and Larsen^25^ found relationships between personality factors and the spontaneous fluctuation rate of electrodermal activity (EDA lability) in a sample of 62 undergraduate students. The respondents filled in personality questionnaires and during this time the skin conductance responses were monitored. They found that the EDA lability strongest correlated with neuroticism. Brumbaugh et al.^26^ measured association between personality (Big Five) and physiological responses in a group of 169 participants, who watched video clips with negatively emotionally saturated scenes. The results confirmed that extraversion and neuroticism were mostly associated with physiological outcomes.

Different kinds of situational performance are often linked to anxiety, which in turn reveals higher physiological responses. Hofmann and Kim^27^, examined the physiological correlates of behavioral inhibition and trait anxiety in speech anxiety. They monitored heart rate and skin conductance level during the experiment on a group of 55 male college students. There were also self-report measures for assessing social anxiety, behavioral inhibition, and trait anxiety. SCL was a better autonomic indicator of trait anxiety than heart rate, while hierarchical multiple regressions revealed that trait anxiety predicted SCL. Neither behavioral inhibition nor trait anxiety predicted heart rate reactivity.

Another study by Wearne et al.^28^ concerned the moderated influence of anxiety sensitivity on the physiological and subjective experience of acute psychosocial stress (Trier Social Stress Test (TSST)) on a group of fifty-eight undergraduate students. The results revealed that heart rate and skin conductance increased in response to the TSST. However, anxiety sensitivity did not moderate any of the physiological outcomes of the TSST.

The linkage between personality, anxiety and responses to different challenging situations has been previously confirmed. In the study by Hidalgo-Munoz et al.^29^ on pilots where the capacity to cope with anxiety during a flight was similar to the prestart situation when the stressful situation occurred, the results confirmed that neuroticism was linked to cognitive and somatic anxiety. Even when the neuroticism scores were moderate, a positive correlation with the pilots’ subjective anxiety was found while they were carrying out the tasks under the social stressor. Other studies are congruent with these findings. In sport and music performance neuroticism and trait anxiety are predictors of anxiety^30,31^. The first one investigated the relationship between personality traits, anxiety and physiological arousal in athletes, and revealed that an athlete’s neurotic personality could influence their cognitive and physiological responses in a competition. Moreover, a study by Dal et al.^32^ revealed that neuroticism could moderate the relationship between anxiety and autonomic activity (respiration) before and during an archery competition.

These studies concerned personality and anxiety in some form of realistic circumstances. However, there is a lack of such studies where the association among these dimensions has been investigated in imagined situations that reflect sport performance familiar to athletes. Therefore, the aim of our study is to understand how individual differences in personality (e.g., neuroticism), general imagery and situational sport anxiety are linked to arousal (e.g., SCL) in situational imagery (as scripted for sport-related scenes).

By relying on the theoretical premises, we constructed a predictive regressive model, based on artificial intelligence methods. We planned to use artificial neural networks to explain the relationship among the tested factors.

Taking into account previous investigations, we linked higher skin conductance level to higher neuroticism (general personality factor) and somatic anxiety (examined in the context of sport), and a general tendency to use imagery in life (H1). Moreover, we explored whether three dimensional aspects of anxiety level in sport diversifies the results (H2). From a practical point of view, we also tested whether different kinds of sport anxiety required a different mental training approach (Q).

## Results

**Figure 1:**
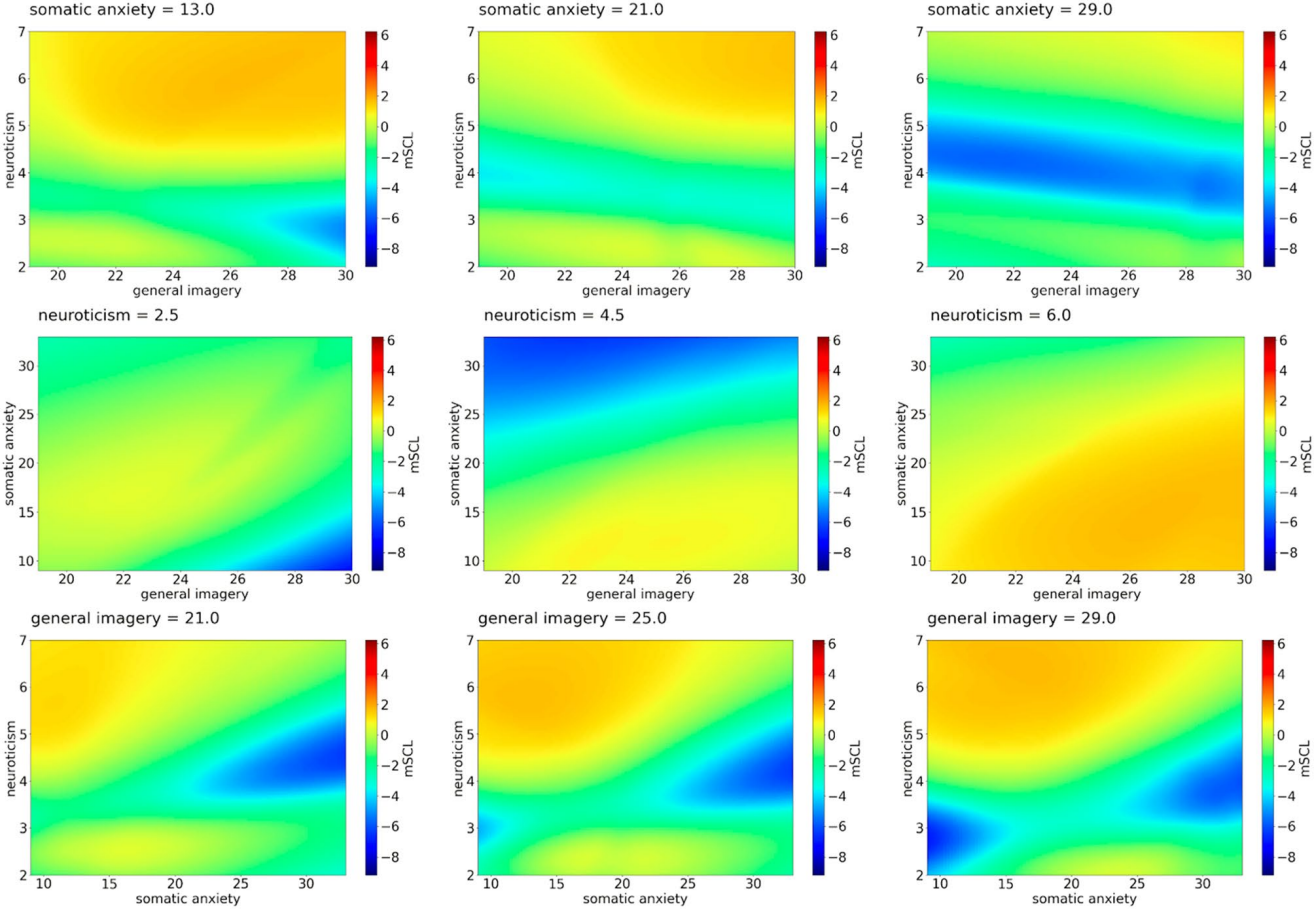
*mSCL* as a function of psychological features—example predictions of Model 1. First row: *mSCL* as a function of *general imagery* and *neuroticism* for three values of *somatic anxiety*. Second row: *mSCL* as a function of *general imagery* and *somatic anxiety* for three values of *neuroticism*. Third row: *mSCL* as a function of *somatic anxiety* and *neuroticism* for three values of *general imagery*. Three chosen values of somatic anxiety, neuroticism, and general imagery represent the middle points of each tercile of these features.

**Figure 2:**
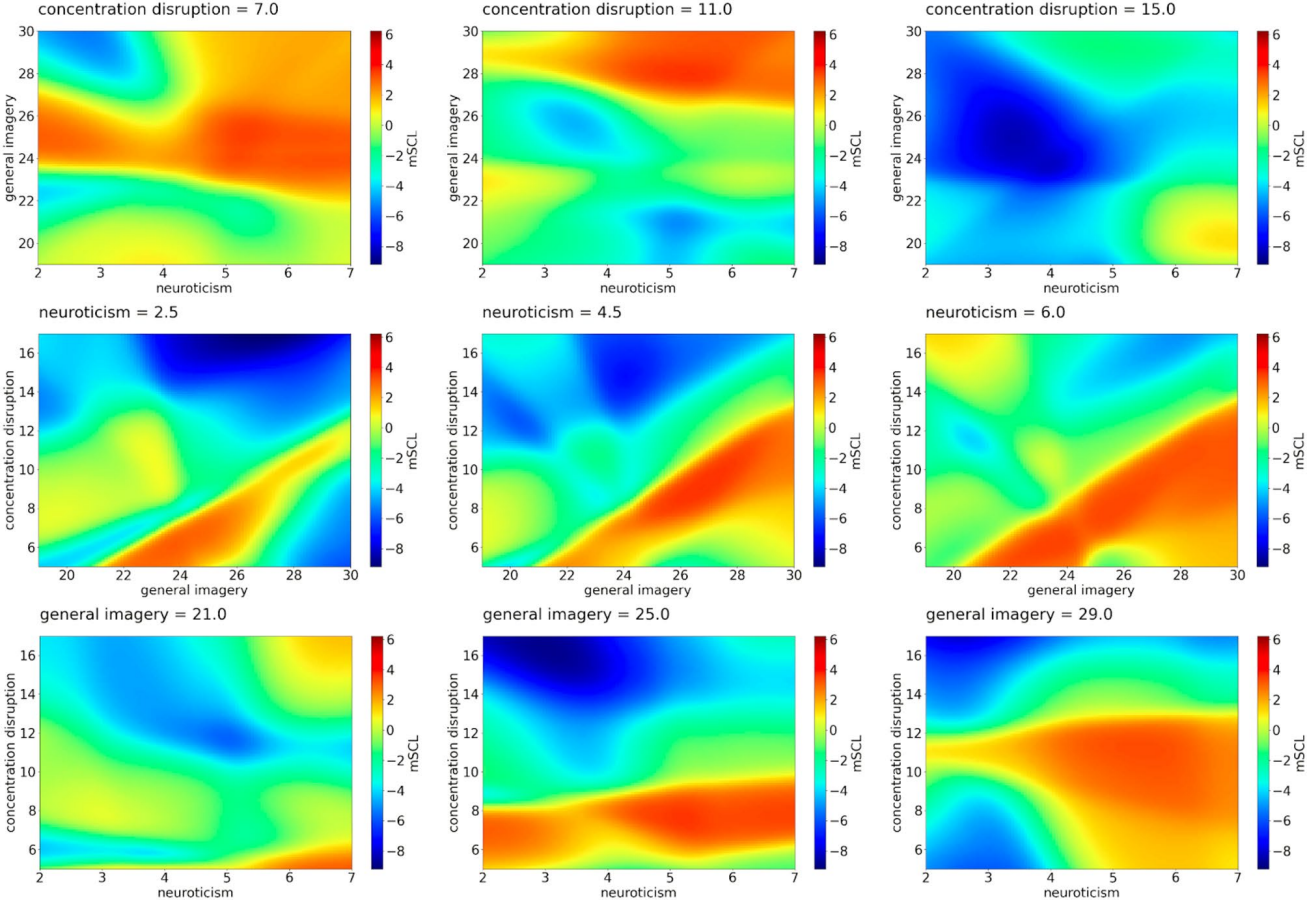
*mSCL* as a function of psychological features—example predictions of Model 2. First row: *mSCL* as a function of *general imagery* and *neuroticism* for three values of *concentration disruption*. Second row: *mSCL* as a function of *general imagery* and *concentration disruption* for three values of *neuroticism*. Third row: *mSCL* as a function of *concentration disruption* and *neuroticism* for three values of *general imagery*. Three chosen values of concentration disruption, neuroticism, and general imagery represent the middle points of each tercile of these features.

**Figure 3:**
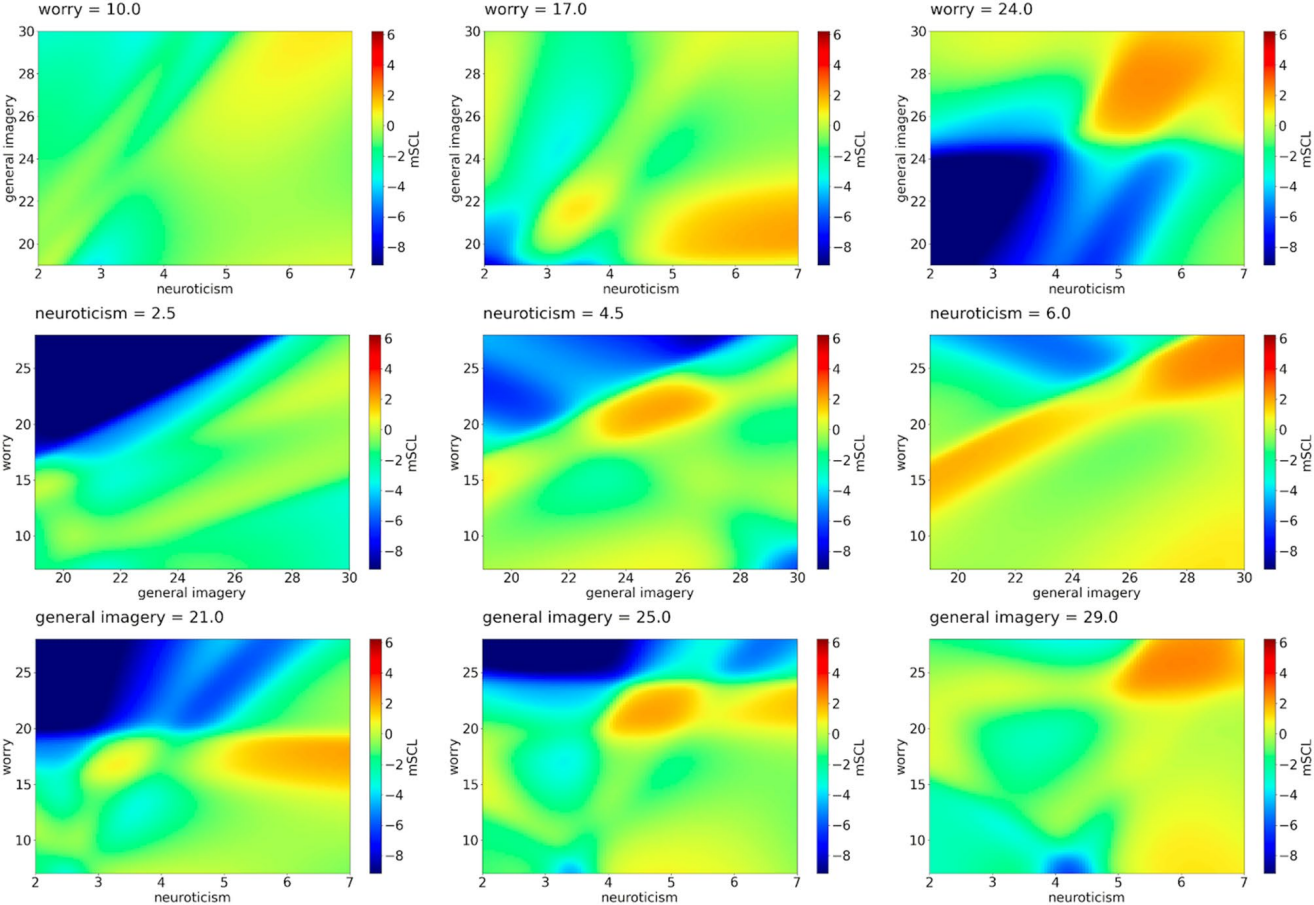
*mSCL* as a function of psychological features—example predictions of Model 3. First row: *mSCL* as a function of *general imagery* and *neuroticism* for three values of *worry*. Second row: *mSCL* as a function of *general imagery* and *worry* for three values of *neuroticism*. Third row: *mSCL* as a function of *worry* and *neuroticism* for three values of *general imagery*. Three chosen values of worry, neuroticism, and general imagery represent the middle points of each tercile of these features.

Machine learning models based on artificial neural networks, trained in a supervised way, have been used to perform predictions of the mean values of normalized SCL (*mSCL*), as functions of psychological factors. Those models are able to reproduce nonlinear relation between predictors and dependent variable. Example results are shown in Figs. 1, 2 and 3. For easier explanation and interpretation of the results, we assigned the term "high" for *mSCL* values greater than 1, "moderate" for those between − 4 and 1, and "low" for values less than − 4. These thresholds represent the 33.3rd and 66.7th percentiles, so each of the chosen groups covered a third of the data, for which the total range changed from -9 to 6. Moreover, we presented the results using colors, where red and orange meant a high level of SCL, yellow and green a moderate level, and blue a low level. A specific legend for the results was included in the figures.

### Somatic anxiety

In the case of the predictors in Model 1, the lowest SCL excitation was noticeable mainly for low and moderate neuroticism, and when somatic anxiety was moderate or high. On the other hand, the SCL excitation remained high if somatic anxiety was low and neuroticism reached high values. Overall moderate SCL excitation was higher when general imagery was higher, except in regions where neuroticism and somatic anxiety were low, or somatic anxiety was high and neuroticism was moderate. The average SCL excitation was also higher when somatic anxiety was lower. This was particularly true if neuroticism was moderate or high. Table 1 shows how predictors from Model 1 change with the SCL level.

**Table 1:** Impact matrix of SCL level for possible configuration of the predictors from Model 1.

SCL	Neuroticism	General imagery	Somatic anxiety
Low	Moderate	All levels	High
	Low	High	Low
	Low	High	High
	Moderate	High	High
Moderate	Moderate	All levels	Moderate
	High	All levels	High
	Low	Low	Moderate high
	Low	Moderate	Low
High	High	All levels	Low
	High	Moderate/high	Low/moderate

### Concentration disruption

In our analysis (Fig. 2) we discovered that high concentration disruption, low neuroticism, moderate, high general were connected to low SCL. The highest SCL was found when general imagery was high, neuroticism was rather high (moderate/high), and concentration disruption was low or moderate. High SCL was noticed when, despite the level of neuroticism, concentration disruption was low and general imagery was moderate. Finally, the next high level of SCL was noticed when neuroticism was high, concentration disruption was also high and general imagery low. Table 2 shows the possible configurations of the predictors from Model 2.

**Table 2:** Impact matrix of SCL level for possible configuration of the predictors from Model 2.

SCL	Neuroticism	General imagery	Concentration disruption
Low	Low	High	High
	Moderate	Moderate	High
	Low	Low	High
Moderate	Low/moderate	Low/moderate	Low
	Low/moderate	Moderate	Moderate
	Moderate/high	Low	Moderate
	Low/moderate	Low	High
	High	Moderate	High
	Moderate	High	High
High	High	High	Low
	High	High	Moderate
	High	Moderate	Moderate
	Low	Moderate	Low
	Moderate	Moderate	Low
	High	Low	High

### Worry

We observed low SCL when neuroticism was low or moderate, general imagery was low or moderate and worry high. Moderate SCL mainly occurred when neuroticism was low or moderate with different configurations of general imagery and worry. High SCL was noticed when neuroticism was high, all levels of imagery and high moderate worry. Table 3 shows how the predictors from Model 3 change with the SCL level.

**Table 3:** Impact matrix of SCL level for possible configuration of the predictors from Model 3.

SCL	Neuroticism	General imagery	Worry
Low	Low	Low/moderate	High
	Moderate	Moderate	High
	Low	Low	High
Moderate	Low	All levels	Low
	Low	Low	Low
	Low	High	Low
	Moderate/high	Moderate	Moderate
	Low/moderate	High	High
	Moderate	Moderate	High
High	High	High	High
	High	Low	Moderate
	High	Moderate	Moderate

In Table 4, the variable importance, calculated using methods described in the references^33,34^, is presented. Clearly, all predictors are of a similar importance in terms of their contributions to the prediction results of each model. Basic metrics of the trained models were also collected. Since the main goal was to achieve good generalization, overfitting prevention was kept a priority over perfect metrics. It was shown that the models were able to reconstruct the desired dependencies with reasonable accuracy.

**Table 4:** Importance of particular predictors, expressed as a percentage contribution to the prediction result for each model, as well as the basic metrics of the trained predictive models.

Predictor	Model 1	Model 2	Model 3
General imagery	26.38%	35.51%	30.74%
Neuroticism	37.09%	28.21%	31.80%
Somatic anxiety	36.53%	–	–
Concentration disruption	–	36.28%	–
Worry	–	–	37.46%
Metrics			
R^2^	1.607	0.508	1.117
RMSE	0.583	0.958	0.799

## Discussion

Our study aimed to understand the role of individual differences in terms of personality traits at a broad (neuroticism) or narrower (anxiety in sport) level, in the general tendency to use imagery and in the psychophysiological response (SCL) during imagery tasks in the sports domain.

Previous research has indeed reported that sports champions had lower levels of neuroticism^35^, thus indicating that intergroup differences among athletes have already been explored. This is also the reason why in our study we focused on individual differences in the context of physiological activation. Our results confirmed the first hypothesis that skin conductance level is positively associated with neuroticism (general personality factor), somatic anxiety (examined in the context of sport), and a general tendency to use imagery in life. In other words, respondents who reveal higher physiological activation during imagery tasks are more likely to experience emotional instability, stress, and anxiety, express more physiological changes as a result of anxiety before and during a competition but also present a higher tendency to use the imagery in daily life. Our findings also confirmed that high neuroticism is linked to high SCL, regardless of the level of anxiety and imagery, which means that neuroticism is mainly the core aspect, and therefore the main predictor of high SCL. Indeed, individuals with higher levels of neuroticism exhibited greater arousal. These results are also in line with previous studies^24,26^, in which neuroticism was connected to greater autonomic arousal responses to emotional scenes. In our study the linkage between neuroticism and SCL was the clearest within the somatic anxiety subscale (the higher the neuroticism, the higher the SCL and the higher the somatic anxiety).

We observed that a moderate level of SCL was mainly linked to a moderate level of at least one predictor. However, the results were not so conclusive. Some lower levels of arousal measured by SCL were linked to high levels of other pre-start anxiety dimensions (concentration disruption and worry) similarly to somatic anxiety, except when neuroticism was high, sometimes alongside high general imagery. Since these athletes showed high sport anxiety levels, we could argue that they were underactivated, or disengaged. This finding may be because the imagined situations were insufficient for them to be physiologically aroused according to Yerkes-Dodson’s first law^36^ or adversely too stimulating and therefore they were disengaged from continuing the task. The last explanation is in line with Pavlov’s protective inhibition theory or Hobfoll's^37^ conservation of resources theories. Therefore, it is worth exploring the role of neuroticism as a general higher reactivity to stimuli alone or with the occurrence of different ranges of general imagery scores. Additionally lower, moderate, and higher levels of anxiety may facilitate or be an obstacle where imagery is concerned. The role of general imagery should be verified.

For the second hypothesis we explored whether the three-dimensional aspects of sport anxiety levels diversify the results. We observed that the highest level of SCL was linked to a moderate/high level of general imagery, a high level of neuroticism and a moderate level of somatic anxiety. Although we did not collect data on the athletes’ performance, we could refer to the framework of the MAP Model^38,39^, which distinguishes four performance categories (i.e., type 1, type 2, type 3, type 4) derived from a hypothesised interaction of optimal/suboptimal and automatic/controlled performance dimensions. Specifically, we could refer to type 2 (optimal-controlled) or type 3 (suboptimal-controlled) performances which are both linked to high energy consumption.

The highest SCL might indicate that the participants were imagining performances in which a high level of control was required, with various possible outcomes. Type 2 is characterized by high performance effectiveness and low processing efficiency, but in type 3 both performance effectiveness and processing efficiency are low. For instance, Bertollo et al.^38^ found that electrodermal activity is more elevated during type 2 and type 3 performances than during type 1 and type 4 performances, and suggested the existence of different mechanisms of energy mobilization and regulation as well as different levels of stress and fatigue. Both mechanisms are associated with an increased sympathetic activation.

Regarding the high level of SCL and neuroticism, we also could ask whether or in what kind of circumstances athletes with that profile are able to achieve the type 1 performance type (high performance with high processing efficiency—flow like state). These results could also be an extension of the MAP model with individual differences included, analyzing the transitions between mental states in different types of performance^40^.

On the contrary, low SCL was observed in the configuration of somatic anxiety with other predictors, mainly when neuroticism was low or moderate. Once again, referring to the MAP model and taking the individual differences perspective, we could argue that when neuroticism and somatic anxiety were low, and general imagery was high, the athletes were calm during the task, thus relaxed and prone to achieving a flow-like state or type 1 performance.

We observed that a moderate level of SCL was related to somatic anxiety, mainly when respondents presented a rather low level of neuroticism, but also with the occurrence of the following conditions: somatic anxiety high/moderate and general imagery low or somatic anxiety low and general imagery moderate/high.

When concentration disruption was taken into account, similarly to somatic anxiety, we observed the highest SCL when neuroticism was rather high (moderate/high), general imagery was high, and concentration disruption was low or moderate. Another profile with high SCL emerged, with all the levels of neuroticism, low concentration disruption and moderate general imagery, which may be indicative of the athletes’ focus on the task. We also observed high SCL when neuroticism and concentration disruption were high and general imagery was low. Athletes with such a profile may be more prone to represent suboptimal performances like type 3 (suboptimal-controlled performance) or type 4 (suboptimal-automatic performance). Our results, as previously mentioned, may be interpreted considering the MAP perspective; however, the individual features may differentiate the SCL signal.

We observed a moderate level of SCL with at least a moderate level in one of the three subscales, mainly neuroticism. When athletes showed low SCL with high concentration disruption, low neuroticism and moderate/high general imagery we might argue that they used imagery. However, in this case, we could not conclude whether they were not focused enough on the task, not interested in taking part in imagining or simply calm.

Considering the worry subscale, similarly to previous anxiety subscales, we observed high SCL when neuroticism was also high. In addition, when neuroticism was high with high levels of imagery and worry, athletes were rather ineffective in their imagery, they used a lot of energy to follow the instructions of the task, or did not control their imagery (type 3 performance in the MAP model). However, when the general imagery and worry were moderate, athletes used some mental skill resources, but we are not aware if they could cope with the task or not (try to do something). Athletes showed high SCL also with high neuroticism, moderate worry, and low imagery, probably because they did not have imagery ability and therefore did not use imagery. When we observed moderate SCL, similarly to previous subscales, rather low or moderate neuroticism was the general tendency. Specifically, when worry was moderate, we also observed moderate levels in the other predictors. Moreover, with low worry, general imagery did not play any role; however, when worry was high then general imagery was moderate or high. These results may indicate that in such circumstances, a general tendency to use imagery could be a useful tool to diminish SCL level.

Considering the relationship between worry and low level of SCL, we observed high worry, low/moderate general imagery, and low/moderate neuroticism, which could be explained using Yerkes-Dodson’s first law^36^ connected with underactivation, low arousal, lack of task concentration and low performance. Low excitable athletes (who show low/moderate neuroticism) should be aware of their need for more arousal to perform optimally.

Overall, we questioned whether participants with the lowest SCL were underactivated or disengaged during the imagery task. Low neuroticism is the most important for low SCL, regardless of other predictors, but low SCL might be caused by the fact that the participants were reacting to imaginary rather than real situations. The aspect of anxiety might indicate motivational aspects in this study, because while they were typically easily agitated in sport situations, here they might have been calm because they were disengaged or not committed enough to be physiologically aroused.

In our study we also tested whether different kinds of sport anxiety would require a different mental training approach (Q); therefore, the role of imagery was also considered. The results were still difficult to interpret, and indeed there is evidence that people with high imagery ability use imagery more often^41,42^, but our results suggest that other factors should be considered as predictors of SCL. Therefore, we distinguish three types of profiles and suggest interpretation based on the characteristics of the predictor:

- does not use imagery: high worry, low or moderate general imagery, low/moderate neuroticism, low mental training skills and predispositions. This means they find it difficult to cope with starts in difficult situations,
- inconsistent use of imagery: high concentration disruption, low neuroticism, higher/moderate general imagery. These athletes use imagery, but they are not able to modulate their level of activation (i.e., SCL) due to a high level of cognitive anxiety,
- uses imagery: low somatic anxiety, low neuroticism, high general imagery, high mental training skills and predispositions. This forms a good basis for optimal concentration along with positive and controlled imagery.

These three profiles could form a realistic hypothesis to test in future research.

A limitation of our study was undoubtedly the rather small sample, but each participant imagined 7 situations, which required complex and time-consuming procedure. Additionally, the sample consisted of athletes who took part in sport competitions. The strength of this study is the length of the scripts, because previous studies often used very short and simple scripts. We also focused on selected individual differences to design the neural network. In the future it is advisable to also check the role of other psychological factors. This is the first study in which not only psychological indices but also physiological biomarkers were used in the neural network method during complex imagery tasks, which undoubtedly constituted the main strength of this work. Due to the complexity of the model, we aimed to analyze predictors of only imagined rather than actual performance, as the latter is typically burdened with many uncontrollable distractors. In the future we would like to enlarge the sample, and include the level of sport experience, the type of sport and gender in the design of the study.

To conclude, in this multidisciplinary work, we found a linkage between neuroticism, prestart anxiety and a general tendency to use imagery with SCL. We extended the aspect of “who (individual)” from the RAMDIU model with psychophysiological parameters and discussed our results in the MAP model framework, in order to shed light on the influence of personality traits in the performance types. From a practical point of view, our results may help athletes, coaches and psychologists to become more aware of the role of individual differences in sport and how to use this information in psychological skills training.

## Methods

### Participants

Thirty participants took part in our study, aged between 14 and 42 years (*M* = 22.93; *SD* = 5.24 (below 20 years—*N* = 5, 20–29 years *N* = 22, 30 and more years* N* = 3)). The majority of the participants (*N* = 25) were emerging adults (emerging adulthood described as typically lasting between ages of 18 and 29^43^). They represented sport experience ranging between 2 and 20 years (*M* = 10.15; *SD* = 4.75 (5 and less years *N* = 7, 6–10 years *N* = 6, more than 10 years *N* = 17) and different sport disciplines (combat sports (*N* = 11), individual sports (*N* = 7), team sports (*N* = 12) and sport levels: international (*N* = 8), national (*N* = 17), regional and local (*N* = 5).

Written consent was obtained from the athletes to participate in the study and personal data protection was properly secured. In case of minors, informed consent was obtained from a parent and/or legal guardian. The investigation followed the ethical principles regarding human experiments as defined in the Declaration of Helsinki, and the study was approved by the local Institutional Review Board (University of Gdańsk, 11/2015).

In our study we used the following questionnaires:

#### Imagery

The Imagination in Sport Questionnaire (ISQ) formulated by Budnik-Przybylska^44^ is a multidimensional 51-item measurement tool with seven subscales, i.e., physiological feelings, modalities, ease/control, perspective, affirmations, visual, and general. The participants imagined themselves before the start in a high-level competition for 60 s and after this task responded to the 51 items assessing the different aspects of the image on a scale from 1 (not at all) to 5 (completely so). All the subscales (except for the “general” subscale) were related to the imagined situation: i.e., situational imagery. The “general” subscale consisted of six questions and was developed separately to assess the general tendency to use imagery. In our study we only used the last scale, as the most stable, the general tendency to use imagery.

#### The sport anxiety scale

*SAS*^45^ adapted into Polish by M. Krawczyński^46^. This is a special sport psychological tool which measures anxiety as a feature; it helps to determine somatic anxiety levels, cognitive anxiety (worrying) and concentration disruption. *Somatic anxiety*—experiencing physiological changes as a result of anxiety—pacing heart, shortness of breath, sweating palms, muscle tension, etc. This kind of anxiety occurs shortly before a start, grows gradually, climaxes at the beginning of a competition and then drops. *Cognitive anxiety* (worrying)—anxious thoughts, worrying, doubts, imagining failure and humiliation. It binds with negative expectations and cognitive concentration on oneself tied to negative self-esteem. *Concentration disruption*—this is an inherent part of cognitive anxiety, and determines a player’s concentration difficulty levels in terms of the start of a competition.

#### BFIS personality

In order to study personality, the Polish version^47^ of the Big Five Inventory-Short (BFI-S) was used^48^. It is a fifteen-item tool with a 7-point scale of answers, where 1 means definitely not, and 7 means definitely yes. This tool is used to measure personality in terms of a five-factor personality theory. Specific subscales measure the following traits: extraversion, Openness to Experience, Agreeableness, Neuroticism and Conscientiousness. Due to its short form, this scale is increasingly used in exploratory research measuring many variables^49,50^.

### Procedure

Subjects listened to a pre-recorded script and were then asked to imagine the scene they listened to for one minute. The study used scripts for 7 sport-related scenes—one test scene: imagining performing 10 squats and 6 situations used from two existing questionnaires on imagery in sport, the Sport Imagery Ability Measure (SIAM) questionnaire in the Polish adaptation and the Imagination in Sport Questionnaire (ISQ). During this task, their body responses were monitored at all times using multimodal device. Six scenes (Fitness Activity, Successful Competition, Your Home Venue, Training Session, Slow Start, Start in a high level championship (precompetitive routine) with a target imagery were presented in random order. There was a brief pause between each block to activate the subject. After the session they were asked to fill out a set of questionnaires—ISQ, SAS, BFIS. As this was the first stage of the Miniatura 5 project entitled “Psychophysiology of the imagery in sport” (2021/05/X/HS6/00459) in this study we have analyzed only the SCL as a measure of arousal during imagery.

### Data preparation

Raw SCL data, collected at a frequency of 40 Hz and expressed in units of microsiemens (μS), were cleaned according to the 1.5 IQR rule^51^. As a result, at the individual level, selected scripts with outlying variance and/or mean values have been excluded from the dataset. The data was then normalized by recalculating against the average value of a 5 s pause before starting each script (pSCL): nSCL = 100 × (SCL − pSCL)/pSCL. As a result, the normalized SCL signal, nSCL, represents the percentage SCL change from the pre-script average. A similar normalization procedure has previously been used elsewhere^52,53^. The test scene (squats), excluded from the main analysis, has been used to calculate the statistical power of the study. The mean difference between mean SCL in the test and the 6 examined scenes (0.023), as well as the standard deviation (0.03) give the standardized mean difference of 0.77. This, with a significance level set to 5% (0.05), gives the statistical power of 91.35%.

The results of the psychological test have also been cleaned, using the same rule as for the SCL data. No outlying values were detected. Data was normalized to the [− 1, 1] range.

### Machine learning models based on artificial neural networks

Models based on machine learning methods make it possible to look for trends and patterns in the data, and to make predictions as a result of supervised learning^54^. In the present work, machine learning predictive models based on artificial neural networks (ANN) were trained in the *supervised* way, in frames of the Tensorflow framework^55,56^ and used for the prediction of physiological response (mean value of normalized SCL, measured during imagery, averaged over all scripts for a particular person, *mSCL*), as a function of selected psychological tests (see Table 5 for models designations).

**Table 5:** Particular models definitions.

Model no	Predictors list	Dependent variable
1	General imagery, neuroticism, somatic anxiety	*mSCL*
2	General imagery, neuroticism, concentration disruption	*mSCL*
3	General imagery, neuroticism, worry	*mSCL*

The choice of three independent models with three predictors each (instead one model with 5 predictors) allows for better overfitting prevention, and facilitates interpretation and visualization of the prediction results, *mSCL.*

**Figure 4:**
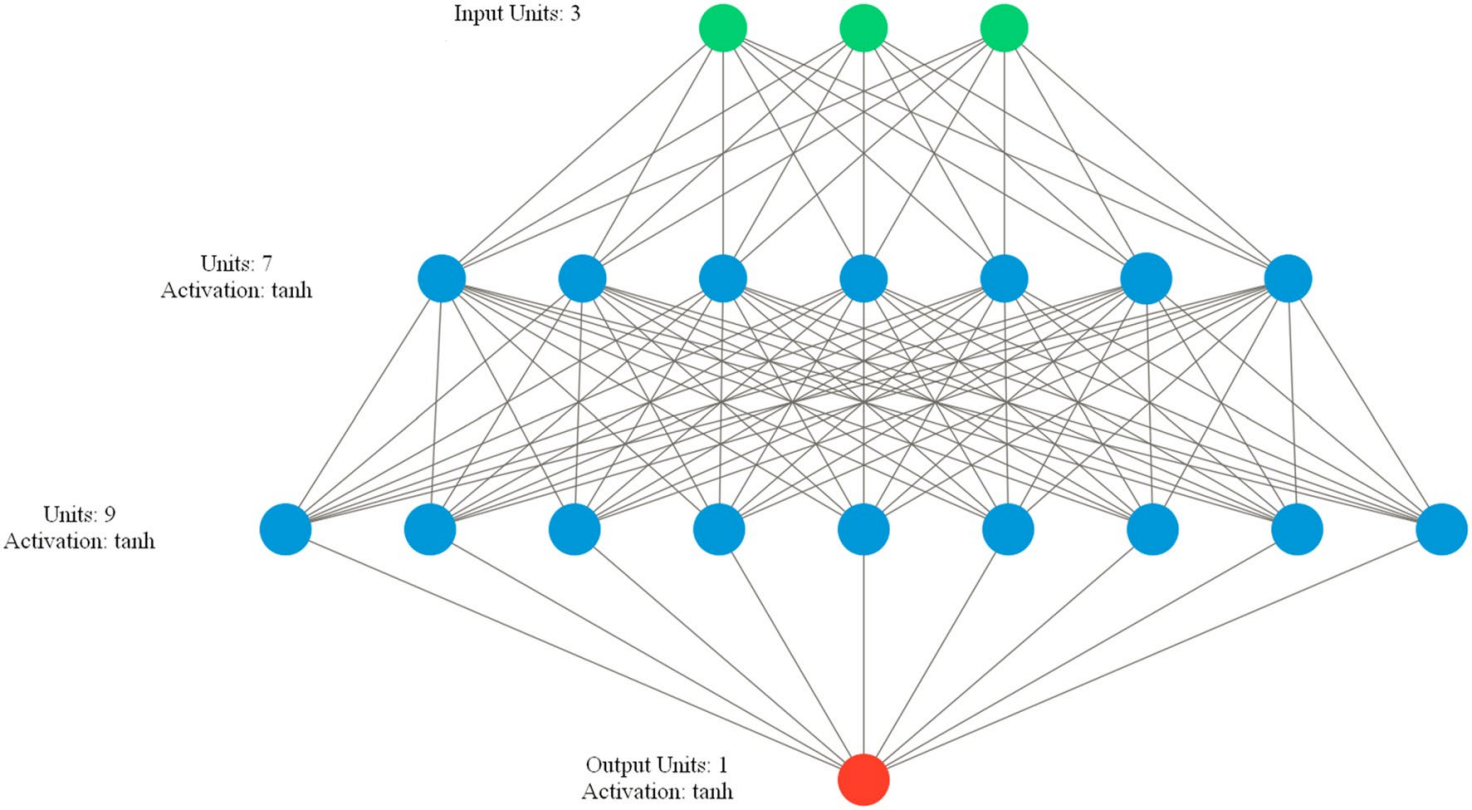
Neural network architecture for the predictive models. The three predictor values are put into the first layer consisting of three input neurons. Then, the computational process continues through the two hidden layers. Finally, mSCL prediction is calculated as the activation of the output neuron.

The ANN architecture used (3–7–9–1 *fully connected* neurons, hyperbolic tangent activations) is presented in Fig. 4. This architecture was found in the *Hyperband* hyperparameters tuning algorithm^57^.

**Figure 5:**
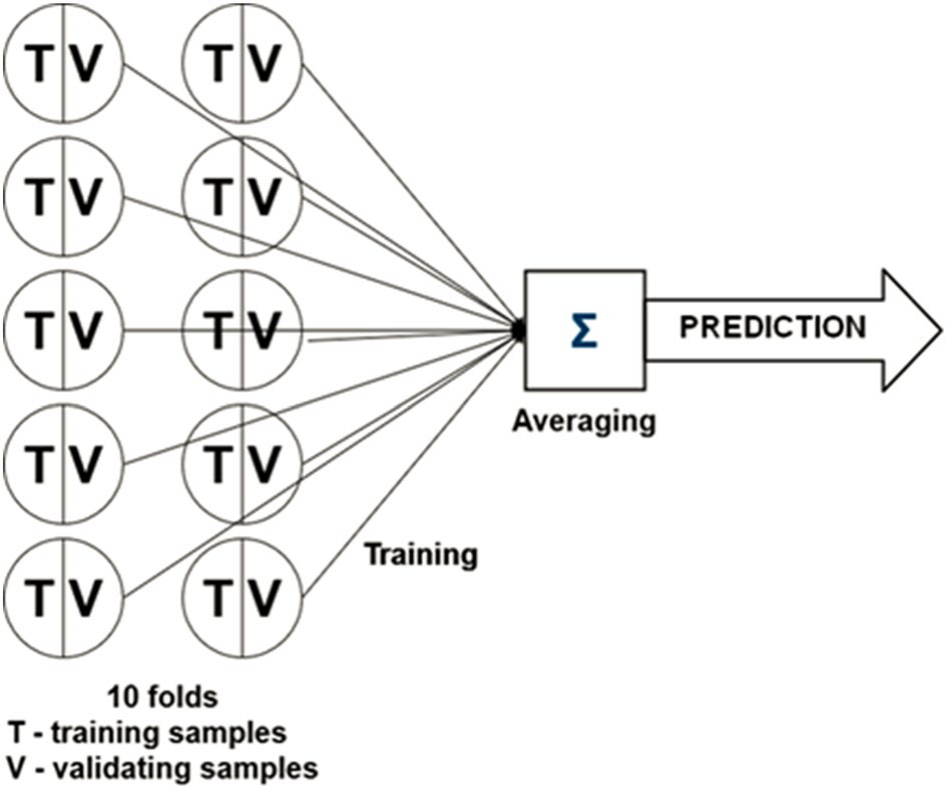
Illustration of the k-fold cross-validation procedure, and construction of the averaged predictive model.

To train the models, the *k-fold* (k = 10) *cross-validation* method^58^ was employed. Except for overfitting prevention^59^, the method allowed us to reduce the size of the validation data set in each model, which is particularly important when the amount of data is limited^60^. In our case, the dataset was randomly divided into 10 *folds*, each of 27 training and 3 validating samples. Each fold was then independently trained using *Adam* optimizer^61^. Additionally, the regularization technique for training of the particular folds was employed: the training was *early-stopped*^62^ when the loss (root mean squared error, RMSE) on the validation samples stopped improving. After that, the final predictive model was constructed by (weighted) averaging predictions from the particular folds (see Fig. 5), where weights were chosen as the inverses of the losses on the data set.

## Data Availability

The datasets generated and analysed during the current study are available in the Bridge of Knowledge Open Data Repository, https://doi.org/10.34808/0qav-2y30.
